# The advantages of TCM in the treatment of gynecologic malignancies

**DOI:** 10.3389/fonc.2025.1570732

**Published:** 2025-09-05

**Authors:** Wei Jiang, Jinghua Yuan, Xinling Zhang, Shuying Zhu, Xiaoping Li

**Affiliations:** ^1^ Department of Gynecology, Shulan (Hangzhou) Hospital, Shulan International Medical College, Zhejiang Shuren University, Hangzhou, China; ^2^ Key Laboratory of Artificial Organs and Computational Medicine in Zhejiang Province, Shulan International Medical College, Zhejiang Shuren University, Hangzhou, China; ^3^ Department of Microbiology Laboratory, Yiwu Center for Disease Prevention and Control, Yiwu, China

**Keywords:** traditional Chinese medicine, TCM gynecology, gynecologic malignancy, therapeutic action, therapeutic status

## Abstract

Gynecological malignancies are characterized by high morbidity and mortality rates. With the development of society, the status of women continues to improve, yet the social pressure they bear increases daily. The incidence rate of gynecological malignancies in the female population has always remained at a high level, and the age of onset has shown a trend of getting younger. Common gynecological malignancies include cervical cancer, ovarian cancer, and endometrial cancer. Alarmingly, over 70% of patients are diagnosed at an advanced stage. This disease is mainly treated through surgery and radiotherapy, but there is still a relatively high recurrence rate after treatment. In recent years, with the development of traditional Chinese medicine (TCM), the advantages of TCM in the treatment of gynecological malignancies have gradually emerged. The entry of TCM into the treatment of Gynecologic malignancies is a tumor treatment method that has received close attention from the international medical community in recent times. TCM can be used throughout the whole process of tumor treatment. Combining Western medicine at different stages of the tumor, or giving different Chinese medicines alone, can minimize the toxic side effects of Western medicine treatment, alleviate symptoms, prolong survival and improve the quality of survival. Therefore, combining traditional Chinese medicine to provide individualized treatment for patients may become a better approach to cancer treatment. This article reviews the status and important role of TCM in gynecological malignancies in the hope of exploring new treatment modalities to mitigate the impact of gynecological malignancies on women’s health.

## 1 Introduction

Gynecological malignancies include endometrial cancer (1), cervical cancer (2), ovarian cancer (3), vulvar cancer, etc. The Yellow Emperor’s Canon of Internal Classic uses terms such as “stony uterine mass” and “metrorrhagia and metrostaxis” to describe gynecological disorders (4). According to traditional Chinese medicine (TCM) theory, the causes of gynecological malignancies lie in the imbalance of qi and blood, dysfunction of internal organs, dietary imbalance, and imbalance of yin and yang within the body (5, 6). This leads to stagnation of phlegm and dampness, blocking the meridians, or the deficiencies of the body, the six exopathogens and toxicity enter the body, resulting in qi and blood stasis (7, 8). The pathogenesis of gynecologic malignancies is related to spleen dampness, liver depression, kidney deficiency, loss of function of the internal organs, and dysregulation of the impulses and ducts (9). Thus, gynecologic malignancies are typically identified as blood stasis and accumulation, liver-qi stagnation, internal obstruction by phlegm-dampness, and positive deficiency of blood stasis (10). The treatment method of TCM is classified as Oriental medicine by the international community and is integrated into biological therapy, which is an important part of the integrated treatment of gynecologic malignancies (11, 12). It has been validated by clinical TCM practitioners over the years and its clinical usefulness has been highly valued by many doctors. Wang et al. found that Guizhi-Fuling Wan exerts anti-tumor effects in ovarian cancer through various mechanisms such as inducing cell death, inhibiting proliferation, and enhancing chemosensitivity (13).

Patients after tumor surgery often experience a decrease in immune surveillance function due to qi deficiency, and residual cancer cells are prone to evade immune clearance (14). TCM enhances the activity of immune cells and improves the ability to recognize cancer cells. The tonifying prescriptions such as Baizhen Decoction and Shenqi Huangqi Capsules contain ginsenosides and astragalus polysaccharides, which can increase the ratio of CD4^+^/CD8^+^ T lymphocytes by 20% (15). By blocking the formation of the tumor-promoting microenvironment, traditional Chinese medicine inhibits inflammatory pathways through multiple targets, downregulates the NF-κB pathway, and reduces the secretion of pro-inflammatory factors such as TNF-α and IL-6 (16). It can also exert its effects by regulating the proliferation and apoptosis of cancer cells (13).

## 2 Therapeutic applications of TCM in gynecologic malignancies

The main treatments for malignant tumors in modern medicine are surgery, radiotherapy, and chemotherapy. Surgical treatment is common in cancer treatment, which achieves the goal of treatment by removing the lesion. Currently, research has proposed ultra-radical exenterative procedures to treat some gynecological malignancies (17, 18). Radiotherapy and chemotherapy involve the use of high-energy radiation or chemical drugs to kill or damage cancer cells. They are crucial for the treatment of locally advanced or inoperable gynecological malignancies (19). Currently, radiotherapy combined with cisplatin concurrent chemotherapy as an adjuvant treatment approach can enhance the overall survival rate of patients (20). These treatments have a decisive role in eliminating the tumor lesions and striving for radical cures (21). However, each of these methods has its indications and limitations, so some tumors can achieve radical cures in the early stages. So far, for advanced gynecological malignancies, single method treatment has side effects and a high recurrence rate in patients. Cancer patients will suffer from the adverse effects brought about by these treatments, such as varying degrees of damage to normal tissues and organs, endocrine disorders, reduced efficacy, and decreased quality of life for the patients (22). Some studies have shown that combined treatments such as surgical treatment combined with radiotherapy and chemotherapy, or radiotherapy combined with cisplatin concurrent chemotherapy, can improve the survival rate of patients (20, 23). Immunotherapy has developed rapidly and, as a new auxiliary treatment method, has provided new opportunities for the treatment of gynecological malignant tumors (24). In addition to traditional treatment methods and immunotherapy, evidence-based TCM treatment is also an effective therapeutic approach. Studies have shown that TCM as an adjunctive therapy for cancer can alleviate symptoms in cancer patients, improve their quality of life, and reduce complications and adverse reactions (25). TCM prescriptions have also been found to have therapeutic effects and reduce adverse reactions in the treatment of ovarian cancer (13).

### 2.1 TCM syndrome differentiation and treatment

TCM syndrome differentiation therapy is not a new treatment method, but an important means of treating gynecological malignant in TCM for many years (26). Dialectical treatment is a characteristic of TCM diagnosis and treatment. By analyzing the symptoms of patients and combining them with TCM theory, Chinese medicine prescriptions are formulated dialectically (27). According to the TCM formula prescribed based on syndrome differentiation, each patient may have different characteristics (28). As a relatively mature treatment method, it is not widely used in cancer treatment, and many patients and doctors are not familiar with this method. In recent years, with the gradual entry of traditional Chinese medicine into the international medical stage, it has received close attention from the medical community.

### 2.2 The principle of treatment based on syndrome differentiation

Diagnosis involves analyzing, summarizing and integrating the information, symptoms and signs collected through the four diagnostic methods of observation, auscultation and olfaction, inquiry and palpation, and transforming them into a certain syndrome. The treatment method based on syndrome differentiation and treatment is the fundamental principle for identifying and treating diseases in TCM, and it is a unique approach to studying and treating diseases. It encompasses the causes, pathogenesis, affected areas, and the relationships among various righteous and evil qi within the body, reflecting the essence of pathological changes at a certain stage of disease development. If treatment is solely based on the patient’s surface symptoms, it would be rather one-sided. Conversely, syndrome differentiation therapy, tailored to each person, offers a more thorough and precise approach, allowing for a deeper insight into the core of the disease (29).

TCM treatment takes a holistic approach, using various methods to address different qualitative contradictions during the disease development process and integrating orderly treatment with holistic treatment (30). Depending on the progression of the disease and the patient’s condition, different treatment strategies will be adopted at different stages. TCM holds a certain position and role in cancer treatment. If combined with traditional treatment methods, it may yield better therapeutic effects (31, 32).

The onset of gynecological malignancies is the result of the interaction of multiple factors such as genetic susceptibility, lifestyle, reproductive endocrine factors and chronic inflammation. This leads to mutations and epigenetic alterations in key genes, disrupting the normal growth, differentiation, repair and death programs of cells. Eventually, cells acquire malignant characteristics and continuously proliferate and spread, forming malignant tumors (33–35). Approximately 10% to 15% of ovarian cancers are caused by mutations in the breast cancer genes BRCA1 and BRCA2.3, which are characterized by multifocality and relatively rapid progression. Mutations and loss of function of TP53 have been found in 60% to 80% of familial and sporadic ovarian cancer cases. These oncogenes activate different signaling pathways, leading to pathogenicity (36). Persistent infection with high-risk human papillomavirus (HPV) is a key factor in the development of cervical cancer, and the integration of HPV DNA into the host cell genome is a crucial step (37). Estrogen-dependent endometrial cancer accounts for 80%. The main driving factor is the long-term and excessive stimulation of endometrial hyperplasia by estrogen, without sufficient progesterone antagonism (38).

Some people believe that TCM can only enhance the immune system and has no effect in fighting against cancer cells. In fact, through scientific formulation and prescription, TCM not only can improve sensitivity and immune function, but also has obvious effects in inhibiting tumor growth and killing cancer cells (39). It also plays an important role in preventing recurrence, preventing metastasis and rehabilitation treatment (40).

In current clinical practice, there is a problem that some physicians have not received systematic specialized training in oncology, which results in their lack of in-depth understanding of the efficacy evaluation system, toxicity reaction spectrum, and dose-effect relationship of Western anti-tumor treatment (41, 42). The limitations of such knowledge reserves make it difficult to establish a scientific framework for evaluating the indications of TCM treatment, and even more impossible to construct a collaborative treatment model integrating traditional Chinese and Western medicine based on evidence-based medicine in order to achieve the dual optimization of therapeutic efficacy and toxicity control (30).

The combination of traditional Chinese and Western medicine treatment enhances the therapeutic effect through the application of long-term comprehensive therapy and evidence-based methods (43). TCM practitioners with a foundation in Western medicine, or Western medicine practitioners with a foundation in TCM, understand the rules of occurrence and development of Gynecologic malignancies in Western medicine, research trends at home and abroad, treatment methods, toxic side effects, and complications, and also have the theoretical knowledge of TCM (41). By applying the diagnostic and therapeutic methods of TCM, corresponding TCM treatments are provided for patients based on different diseases, their development and changes, as well as different treatment stages (44).

Although the Western medicine treatment for tumors is effective, it often brings certain side effects to patients and may even cause new damage to their bodies. While modern medicine can mitigate some toxic side effects of treatments, most patients will still face various syndromes as a result. Chinese herbal medicine is crucial in this treatment process (42). TCM practitioners with experience in oncology treatment are adept at minimizing those adverse effects, resulting in quicker relief of clinical symptoms, better quality of survival, and longer survival periods (45).

### 2.3 Integration of traditional Chinese and Western medicine for resource conservation and promoting development

If a patient is treated with TCM in conjunction with Western medicine, it is possible to minimize the toxic side effects and maintain the body in good condition to complete the treatment on schedule (41, 46, 47). This will lay the foundation for the patient’s therapeutic effect and long-term healthy survival, reduce additional medical expenses, lower medical costs, reduce the medical burden and financial expenses, and avoid the tragedy of poverty and bankruptcy due to cancer (44). Furthermore, TCM therapy for tumors should not be seen as a substitute for Western medicine or used only when other treatments are no longer viable. There are many different methods of TCM treatment. Oral medicine is the mainstay of TCM treatment, as it is individualized and treatment based on syndrome differentiation. According to each person’s physique, different prescriptions are used for the same case, and the same prescription can cure different diseases. At the same time, there are also a series of methods such as oral medicine, intravenous drip TCM, acupuncture, qigong, food therapy, and external application, which together constitute the comprehensive treatment of TCM and have great advantages in the prevention, treatment, and prevention of tumors (48, 49). Only Chinese herbal medicine can be used from the beginning to end of tumor treatment, and if treated properly, patients can significantly reduce their symptoms, prolong their survival and improve their quality of life.

Chinese herbal decoction plays an important role in cancer treatment, and many types of decoctions can be used in the treatment of gynecological malignant tumors (50, 51). As a commonly used medicine in TCM, ten significant tonic decoctions has been used to treat various diseases (52). Besides, it also plays an important role in anti-tumor and regulating immune response (53). It reduces the side effects of chemotherapy, radiation, and surgery and can prevent cancer metastasis (54, 55). Buzhong Yiqi decoction has also been found to have strong immunomodulatory and anticancer effects (56). It has significant chemo-preventive effects on ovarian cancer cell lines by inducing cell apoptosis or inhibiting cell cycle (52, 57, 58). Other herbal medicines such as astragalus decoction (59) and sho-saiko-to (60) are also fully used in the treatment and prognosis of cancer and play an important role in the treatment of TCM (61, 62) (**Table 1**).

**Table 1:** The application of Chinese herbal decoction in the treatment of gynecologic malignancies.

Common name	Composition	Biological activity	Clinical evidence of anticancer activity	References
Ten significant tonic decoctions	Ginseng radix, Astragali radix, Radix angelicae, Rehmanniae radix, Atractylodis lanceae rhizoma, Cinnamomi cortex, Poria, Paeoniae radix, Rhizoma Ligustici and Radix Glycyrrhizae	Antitumor immunomodulation	①Alleviating hematotoxicity among breast cancer patients undergoing chemotherapy; ②Slow down the process of hepatocarcinogenesis and improve hepatic recurrence-free survival through the inhibition of Kupffer cell-induced oxidative stress in patients with HCC; ③Regulate T cells through decreasing Foxp3+ Treg populations in advanced pancreatic cancer patients	(18–21)
Buzhong Yiqi decoction	Pinellia tuber, Scutellaria baicalensis, Zingiberis rhizoma, Zizyphi fructus, Coptidis rhizoma, Glycyrrhiza radix, Panax ginseng	Antitumor immunomodulation	①Prevent surgical stress-induced immunosuppression; ②Improve cancer-related fatigue and quality of life in cancer patients; ③Reduce side effects such as leucopenia, intestinal damage and fatigue induced by radiation or chemotherapy	(18, 22–24)
Sho-saiko-to	Bupleurum falcatum, Scutellaria baicalensis, Panax ginseng, Zizyphus jujube, Pinellia ternate, Zingiber officinale, Glycyrrhiza glabra	Antitumor, anti-inflammatory antioxidant, Immunomodulation, hepatoprotective, anti-hepatic fibrosis	①Prevent the development of HCC from hepatitis C virus-associated liver cirrhosis (HCV-LC) in the HCV-LC patients; ②Decrease the incidence of stomatitis in cancer patients undergoing chemotherapy	(18, 26)
Astragalus decoction	Scutellaria baicalensis Georgi, Paeonia lactiflora Pall, Glycyrrhiza uralensis Fisch, Ziziphus jujuba Mill	Antitumor anti-inflammatory	①Provide a safe and feasible salvage therapy combined with capecitabine after gemcitabine failure for advanced pancreatic cancer	(18, 25, 27, 28)

## 3 Clinical application of TCM in multimodal treatment of gynecologic malignancies

### 3.1 The role of TCM in oncological surgery

**Figure 1:**
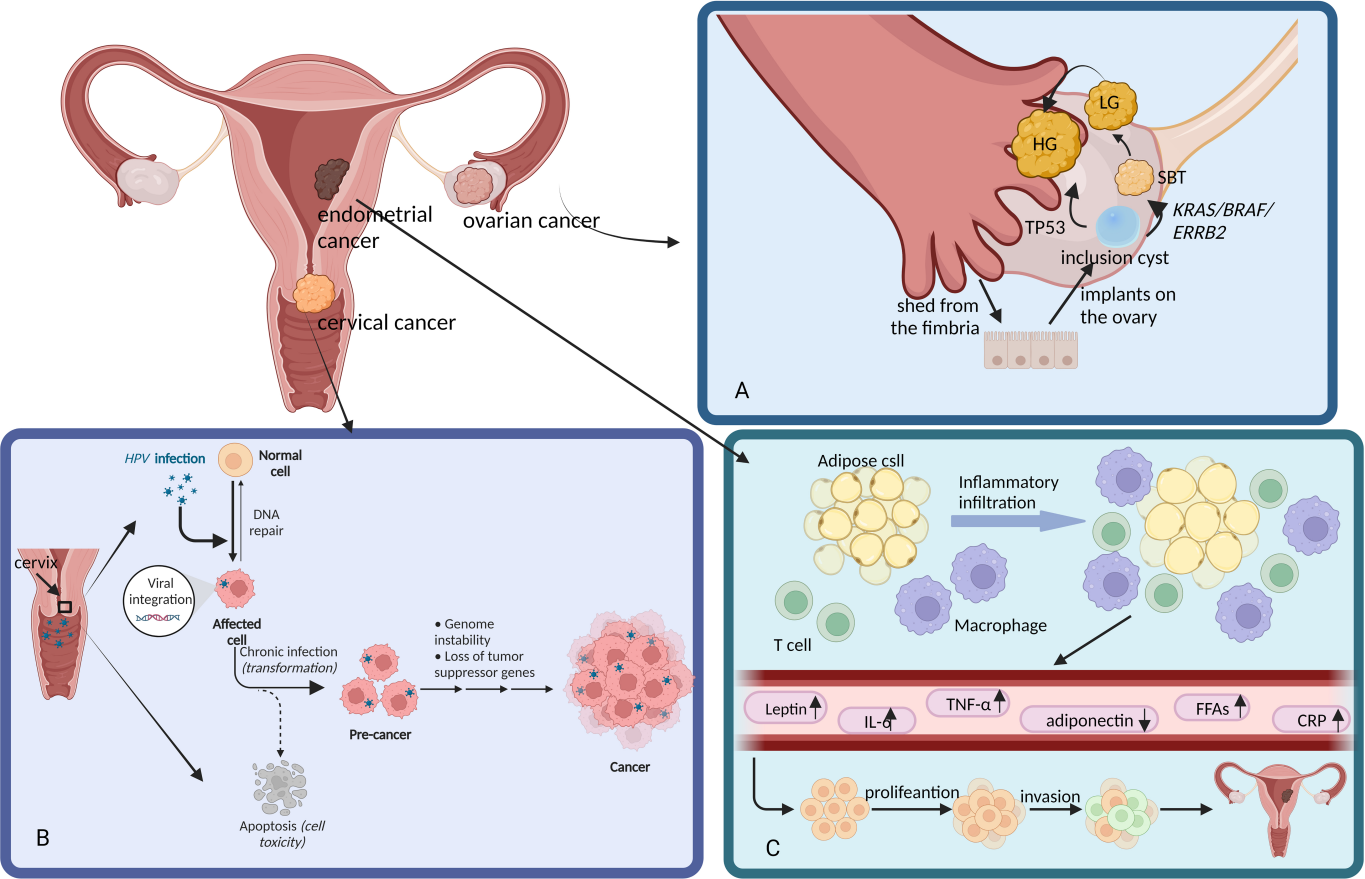
The distribution and pathogenesis of three gynecological malignancies *in vivo*. This figure was drawn by Figdraw. **(A)** Normal tubal epithelium is shed from the pubic hair and implanted into the ovary to form an inclusion cyst. Low-grade serous carcinoma (LG) is dependent on KRAS/BRAF/ERRB2 mutations and usually develops from serous borderline tumors (SBT), which in turn stem from serous cystadenoma. High-grade serous carcinoma (HG) is dependent on TP53 mutations and can develop from LG. **(B)** Cervical cancer is caused by HPV virus infection, HPV into the human body after invasion of the human mucosa or the basal layer of the skin cells, and continue to replicate and proliferate. **(C)** Adipocytes are infiltrated by T cells and macrophages, it can secrete leptin, IL-6, TNF-α, FFAs, CRP and other biologically active adipokines, which lead to endometrial hyperplasia and cancer.

The first choice of treatment for gynecological malignancies is surgery. Cervical cancer is caused by the HPV virus, infiltrating basal cells (63). Endometrial cancer is triggered by excessive estrogen stimulation, leading to malignant transformation of the endometrial glandular epithelium (38). The early stage of ovarian cancer is formed when the epithelium of the fallopian tube is shed and implanted in the ovary and is subject to constant mutation (**Figure 1**) (64). These tumors have different pathogenesis and distribution and can be surgically removed in their early stages. However, gynecological malignant tumors are prone to recurrence after surgery. Even with radical or extensive resection, some patients will still experience recurrence and metastasis. This is because surgery can only remove visible tumors and cannot eradicate latent lesions (65). Furthermore, before the primary lesion is removed, tumor cells may have already entered the blood circulation and spread. Although most of them are cleared by the immune system, a small number of residual cells can proliferate under suitable conditions, becoming the source of recurrence (66). In the comprehensive treatment strategy for tumor surgery, TCM plays a significant synergistic role (25). During the preoperative stage, administering appropriate TCM based on syndrome differentiation can effectively regulate the patient’s constitution and enhance the body’s tolerance to surgical trauma (67). For instance, methods such as nourishing qi and blood, strengthening the spleen and kidney can improve the patient’s preoperative weakness or related symptoms (41). In the postoperative stage, timely intervention with adjuvant TCM therapy can significantly reduce tissue damage caused by the surgery itself and effectively lower the incidence of common postoperative complications, such as controlling non-infectious fever and preventing or correcting postoperative anemia (68). TCM treatment can also promote tissue repair and healing of surgical wounds, and aid in the recovery and reconstruction of damaged organ functions (69). It is conducive to post-operative rehabilitation, reduces recurrence or metastasis, prolongs survival, and at the same time creates better conditions for patients to receive radiotherapy (70).

Surgical resection combined with TCM therapy has been gradually applied to the treatment of tumors. In addition, western medicine and integrated Chinese and western medicine are also important treatment methods, and different treatment methods can achieve different effects. Ovarian cancer (71), endometrial cancer (72), and cervical cancer (6, 73) are the three kinds of gynecological malignant tumors with the highest incidence. Without timely intervention and treatment, their incidence can reach 6~15/100,000, and the mortality rate can reach 2~8/100,000, seriously affecting women’s health. The remission rate and survival rate achieved by the treatment of TCM (3, 74, 75), western medicine (76–79), and integrated Chinese and western medicine are different (80). Only by choosing the appropriate treatment method, it can better improve the survival and prognosis of patients (**Table 2**).

**Table 2:** Effect of different treatment methods on three kinds of gynecologic malignancies.

Gynecologic malignancies	No drug therapy		Traditional Chinese medicine		Western medicine		Combine TCM and western medicine		References
	Incidence	Mortality	Remission rate	Survival rate	Remission rate	Survival rate	Remission rate	Survival rate	
Ovarian cancer	6.52/100,000	4.17/100,000	75%	30%~50%	47%	20%~30%	83%	40%~60%	(3, 31, 34, 35)
Endometrial cancer	11.99/100,000	1.9/100,000	65.8%	50%~70%	60%	40%~60%	44.5%	80%~90%	(32, 36–38)
Cervical cancer	15.6/100,000	8.8/100,000	81.6%	31%~67%	46.7%	60%~90%	88.6%	70%~90%	(5, 33, 39, 40)

### 3.2 The role of TCM in chemotherapy for gynecological malignancies

Chemotherapy, a treatment for malignant tumors, has received increasing attention in recent years. It can injure tumor cells systemically and is particularly therapeutic for tumors that are potentially or have metastasized distantly. Chemotherapy plays a pivotal role in the treatment of ovarian cancer, choriocarcinoma, and malignant staphyloma (81). Choriocarcinoma and malignant staphyloma can be cured by chemotherapy alone without surgery, even highly malignant epithelial malignancies can be cured at an early stage with less extensive surgery with adequate chemotherapy and preservation of fertility. When systemic chemotherapy is combined with surgical removal of local lesions, it can eliminate as many residual tumor cells as possible with minimal burden, reduce the recurrence rate, improve the cure rate, and prolong survival (82). The standard treatment for ovarian cancer is surgical removal and platinum-based chemotherapy (83). The goal of early surgery is to completely remove the visible tumors (83). After the surgery, patients will receive platinum/paclitaxel-based treatment as the first-line chemotherapy (84). This approach has achieved some success in treatment and has prolonged the survival of patients to a certain extent (82). Advanced cancers, whether ovarian, uterine, or cervical, can be treated with chemotherapy as one of the treatment options, in conjunction with other methods (85). Chemotherapy is considered in terms of the biochemical mechanism of the drug, pharmacokinetic relationship, tumor cell proliferation kinetics, and drug toxicity. Chemotherapy drugs have difficulty in distinguishing the metabolic differences between tumor cells and normal cells. While killing cancer cells, they also damage normal tissues, resulting in severe toxic side effects such as gastrointestinal reactions, bone marrow suppression, immune suppression, and infections (86, 87). These side effects not only affect the quality of life of patients but may also interrupt the treatment. Although Western medicine has drugs for anti-nausea and other purposes to alleviate some of these side effects, the effects are limited, they have their own side effects and are costly. Combining TCM treatment can effectively reduce the main toxicities of chemotherapy (such as protecting bone marrow hematopoietic function and reducing gastrointestinal reactions), improve patients’ tolerance to chemotherapy, treatment completion rate and quality of life, and play an important synergistic and enhancing role (22).

Study by Zhang Hong et al. included 50 patients in the chemotherapy interval period and randomly divided them into two groups (25 cases each). The treatment group was given modified Shiquan Dabu Tang combined with other treatments, while the control group only received follow-up. After 3 courses (20 days per course) of treatment, the self-assessment scores of QLQ-C30 and traditional Chinese medicine symptoms in the treatment group significantly improved (P < 0.01), while there was no improvement in the control group (P > 0.05); and the differences between the groups were significant (P < 0.05 or P < 0.01) (88). This indicates that modified Shiquan Dabu Tang can significantly improve the quality of life of patients with gynecological malignant tumors during chemotherapy.

The QLQ-C30, as a quality of life tool for international clinical trials in oncology, consists of nine multi-item scales, five functional scales (physical, role, cognitive, emotional and social), three symptom scales (fatigue, pain, nausea and vomiting), as well as a global health and quality of life scale (89). It is reliable and effective in measuring the health quality of life of cancer patients (90).

Pan Yuzhen et al. found that the combination of Xiaojie Decoction and cisplatin had a stronger inhibitory effect on the proliferation and induction of apoptosis in ovarian cancer drug-resistant cells (CAOV3/CDDP) compared to cisplatin alone, indicating that the two have an enhancing and sensitizing effect. The mechanism may be related to the induction of tumor cell apoptosis by Xiaojie Decoction (91). In addition, for patients in the chemotherapy remission period, supplementing with traditional Chinese medicine that enhances immune function can help promote recovery.

### 3.3 The role of TCM in radiotherapy for gynecological malignancies

The high-energy rays used in radiotherapy not only kill cancer cells but also damage normal tissues, thereby affecting the therapeutic effect (92). Clinical studies have shown that the combined use of appropriate TCM can not only effectively alleviate the short-term toxic side effects and long-term sequelae of radiotherapy as well as radiation damage, but also enhance the efficacy of radiotherapy itself (93, 94). For instance, yin-nourishing and heat-clearing TCM can alleviate the dry mouth and sore throat caused by radiotherapy (95); blood-activating and stasis-resolving ones can prevent radiation-induced fibrosis and promote tumor regression (96); and heat-clearing and detoxifying ones can counteract the heat-toxin damage related to radiotherapy (96). This is an important strategy for comprehensive treatment of malignant tumors.

### 3.4 The role of TCM in advanced gynecologic malignancies

At present, about two thirds of cancer patients are in the middle and late stage at the time of diagnosis, and most of them have lost the opportunity of western medicine treatment. Even if radiotherapy is applied to shrink the local tumor, it does not prolong the life of some patients. For some cancer patients, their death is not caused by the primary disease itself, but rather by the toxic reactions resulting from radiotherapy and chemotherapy; while for others, they lose the opportunity for standardized treatment due to their inability to tolerate the treatment-related adverse reactions or because of economic constraints (97). TCM can sometimes offer a ray of hope for mid and late-stage cancers. This is because the principle of TCM in fighting tumors is to support the righteous and dispel the evil, adjust yin and yang, and take care of the whole body (69). While attacking the tumor, more attention is paid to supporting the body’s positive energy, improving the immune system, and relying on its anti-disease ability to fight against the tumor. On the one hand, it can make the tumor grow slowly or shrink, on the other hand, it can adjust the body to adapt to the new internal environment, reduce the damage caused by cancerous tumors to the body, and appropriately extend the survival period. In some cases, the tumor has shrunk and the quality of life has improved, while in others, although the tumor has not shrunk, the patient can move freely and live on his own, surviving with the tumor for a long time (98). The core advantage of the TCM diagnosis and treatment system lies in its individualized treatment dimension. Regardless of the disease stage or severity, it can achieve optimal clinical efficacy through evidence-based therapies and overall regulatory mechanisms (66).

### 3.5 The role of TCM in the rehabilitation of gynecological malignancies

The occurrence of tumors is a local manifestation of systemic disease. Therefore, patients cannot be completely “cured”, even after radical surgery, radiotherapy and chemotherapy. In modern medicine, the best treatment that can be achieved is “complete remission,” not “cure.” According to the basic theory of TCM, the basic balance of Yin and Yang is achieved. Once the recipient is affected by various pathogenic factors inside and outside, the imbalance of Yin and Yang, the tumor is easy to relapse. Therefore, in the recovery period from the disease, TCM can be used to improve the patient’s autoimmune function and anti-cancer ability, maintain the stability of the internal environment of the body and play a role in dispelling evil and strengthening health (99). At the same time, we must pay attention to regular reviews. Regular review is generally recommended within 2 years after surgery, every 3 to 6 months, and 3 to 5 years after surgery, every 6 months to 1 year. The balance of Yin and Yang is regulated through Chinese medicines and regular review, to prevent disease and reduce the possibility of recurrence (100).

## 4 Summary and outlook

Modern medical treatment of tumors pursues “tumor-free” and the size of the tumor after treatment is the standard for solid tumors. In contrast, the treatment of tumors in TCM is based on the identification and treatment of tumors as the pivotal point, and it is proposed that survival with tumors is possible (101). It emphasizes the improvement of clinical symptoms and quality of life and adopts different treatments for different diseases and different manifestations of patients at different stages of tumor. Enhancing the treatment of gynecological malignancies requires integrating various methods from both TCM and Western medicine, leveraging their strengths, and combining them effectively. TCM has been applied in the treatment of malignant tumors for over two thousand years and has accumulated many valuable experiences with unique treatment methods and clinical efficacy, especially in recent years there have been more innovations and developments in progress. TCM and modern medicine interpenetrate, and complement each other’s strengths and complement each other’s advantages, it plays an important role in complementing surgery, radiotherapy, and chemotherapy. In addition, TCM can also inhibit the development of tumors, improve the quality of life of cancer patients, and extend their survival time. It also plays a role in preventing the recurrence and metastasis of tumors.
